# The Kings and Slaves of Business

**Published:** 1874-12

**Authors:** James Parton


### Our Exchanges.

*NEW YORK HERALD OF HEALTH—December*, 1874.

THE KINGS AND SLAVES OF BUSINESS—*By James Parton.*—The aristocracy of a country are they who wield its resources. In all the tribes and nations of which we have any knowledge, we find the strong forming a class by themselves—usually in the older countries a king and nobility, descended from the ancient strong, who banded together to defend the State against foreign foes and internal anarchy. But, in our modern world, war having become the occasional instead of constant necessity of nations, the soldier recedes, while the man of business comes to the front. Nobilities decay. The great houses of a country are mercantile, not feudal. If we by chance hear the names of York and Lancaster, we do not think of white rose and red, or cracked crowns on bloody fields; but ask, perhaps, whether they are in cotton or in sugar, whether they are iron men or pork men. And this is not retrograde, as some would have us think, but advance, signal and glorious advance. In a country governed by law and devoted to the arts of peace, men of business are the natural chiefs of the community. I have visited more than once the frontiers of the United States, and observed the new settlements and their rising towns, where everything arranges itself in the natural way. In the dense, magnificent forests of Wisconsin, where timber is the staple commodity, what is it that the settlers want, and must have, and can't get? A saw-mill. Who builds it?—the strong few. And around the saw-mill, which is the life and opportunity of the place, the crowd gathers, as in the olden time the retainers nestled in the shadow of the lord's castle. But on the rolling prairies of Minnesota the need of the people is the steam elevator, which towers aloft, visible for many a mile, the center of the grain growing system. On a fine day in winter, as far as the eye can reach you see the sleighs heaped with sacks, and the farmer in his cap of buffalo skin, with his wife or daughter by his side, moving at a gentle trot toward this tower of refuge, which sucks up the grain on one side, and on the other it pours it out into the freight car, while inside is the office where the farmer gets his money, and near by are the stores in which his wife can spend it. These giant structures belong to the strong men of the prairie world, the successors of the ancient nobles. It is not the effeminate idlers who have inherited the names and titles of the old dukes and barons that are their true successors, but men who fill the place in the modern world that dukes and barons did in the ancient.

AN ALLY GAINED.

During the last eighty years men of business have gained an ally that has prodigiously increased their power—the steam-engine—which to-day has done about three times as much work as the whole working population could have done without its assistance. Hence the magnitude of fortunes and the vast, far-reaching enterprises now conducted by individuals. "As rich as Crœsus" is a proverb all over the civilized world, and Crœsus really appears to have had a good deal of gold lying about in his house. Herodotus, to whom he owes his immortality, records that he rewarded one of his generals by letting him go into his treasury and take as much gold as he could carry away on his own person. The general improved his opportunity. Crœsus, too, was a gentleman capable of propitiating Apollo with an offering of three thousand oxen, and a solid golden lion as large as life. But he was a king who had thirteen nations to plunder. The great fortunes of Rome, of which Pliny speaks, were also the spoils of conquered provinces—fortunes accursed in the getting, the holding and the spending. In our happier day, plain men of business wield vaster resources, and direct far-reaching enterprises which have no draw-back of suffering or evil, but benefit every person affected by them. And as the Crœsus with his heaps of gold, there is a carman in New York, at the corner of Broad and Wall streets, who often in one day carries three tons of gold upon his cart; but it is not to Apollo's shrine that he takes this interesting metal. It is surprising how small a matter a million dollars appears in the midst of the immense operations of our day.

A PERFECT BANKER.

The last time I was in Wall street I heard a noted banker say, with great earnestness of manner: "My ambition is to be a perfect banker. I would rather retire from business with only a single million, with the reputation of a great banker, than to be ever so rich without that reputation."

"Only a single million." You cannot imagine how small that million seemed when he uttered these words—a kind of honorable poverty, a Diogonese tub with a man inside of it. You thought of his children boasting that their parents were poor but honest. I can well believe the familiar story of John Jacob Astor saying that "a man who is worth $250,000 is just as well off as though he were rich." The mind easily becomes habituated to these magnitudes. Thomas Brassey, the English railroad contractor, had at one time eighty thousand men in his employ, whose wages came to about half a million dollars a week, and they were at work in eight countries—England, France, Spain, Italy, Venezuela, Canada and Australia. He wrote a note one day ordering ten thousand wheelbarrows and three thousand wagons. In the course of forty years he helped to construct one hundred and fifty railroads, and he did all this with less fuss and worry than many a man experiences in running a peanut stand. We have a greater than Thomas Brassey in Cornelius Vanderbilt, who, at eighty, quietly makes up his mind that the time has come for us to have a four-track railroad from New York to Buffalo. Some one said to him, "I don't know of any four-track roads." "I don't," said he; "but the Central must have two more tracks, and I hope to live to see them laid." And probably he will. The care of an estate of forty millions of dollars and the management of all his railroads only occupy him two hours a day, and during that short time he does not appear to be very busy. By three o'clock he is usually on the road, erect, magnificent, driving a pair of fast horses, in open wagon, unattended.

There are higher spheres of labor than making railroads, in which the great resources of our kings of business are employed with widely beneficial effects of another kind. Many of you have seen in Broadway the ornate and spacious publishing house of the Appletons. Ascend to the fourth story and you find an apartment about fifty feet square, fitted up with desks and pigeon-holes, and occupied by about thirty or forty gentlemen, writing, studying, proof-reading, most of them advanced in age, and several venerable, with gray hairs and great names. This is the working room for the writers of Appleton's Cyclopedia. In this room the whole mass of human knowledge on all subjects and sciences is gathered, and re-stated in such a manner as to make it conveniently accessible to all the American people who can read.

And this work went on for two years before the first volume was given to the printer. No individual, no college for the learned could produce such a work. It becomes possible only by the union of a large body of learned persons, with the capital, enterprise and skill of men of business. Similar works are executed by the Harpers and others, and I do not doubt that the system is destined to achieve still more remarkable triumphs in the future.

And now the question occurs, who are these uncrowned monarchs of the modern world? What are they? Where do they originate? By what means do they accumulate these great resources? In what respect do they differ from the men who attempt a similar career and fall out of the course disabled and defeated? Is it their virtue or the want of virtue that gives them success? These are interesting questions, because these men are the masters of our modern world. They carry the purse and pay everybody, and it is apparently a law of nature that the hand which signs the check wields the power.

As a rule, these kings of business begin life near the bottom of what is called the social scale. They generally begin very poor. Crabb Robinson records that the late Lord Southampton applied to the Bishop of Llandaff for advice how to bring up his son so that he would get forward in the world.

"I know of but one way," replied the Bishop; "give him parts and poverty."

"Well, then," said the father, who was noted for his free living, "if God has given him parts, I will manage as to the poverty."

Another English nobleman, Lord Derby, mentioned a curious circumstance in point the other day. At a gathering in Australia four persons met, three of whom were shepherds on a sheep farm. One of these had taken a degree at Oxford, another at Cambridge, and the third at a German university. The fourth was their employer, a squatter, rich in flocks and herds, but scarcely able to read and write, much less to keep his own accounts. But I venture to say the squatter knew sheep, knew land, knew markets, knew wool. It is an unquestionable fact that in both Australia and America these founders of huge businesses are, as a rule, unlettered men, whose college was a rough, hard, long grapple with material things.

It is also true that they usually take hold, very early in life, of some plain work that lies under their noses, and keep to that until it issues into something large and magnificent. The wealthiest and the most powerful house of business in Europe is that of the Rothschilds, who provide for belligerent kings the sinews of war. The founder of the house, one hundred years ago, was a boy in his father's money-changing office at Frankfort-on-the-Main. He discovered that some of the coins in which his father dealt had an artificial value as specimens, and he used to spend the first day of the week—the Jew's idle day—in picking out such coins from the mass to sell to collectors. That was the beginning of his career. He took hold, you will observe, of the work that lay most obviously before him.

EXAMPLES OF HOW MEN HAVE BECOME RICH.

Isaac Rich, who left a million and three-quarters a year or two ago to found a college in Boston, began business thus: At eighteen he came from Cape Cod to Boston with three or four dollars in his possession, and looked about for something to do, rising early, walking far, observing closely, reflecting much. Soon he had an idea: he bought three bushels of oysters, hired a wheelbarrow, found a piece of board, bought six small plates, six iron forks, a three-cent pepper box, and one or two other things. He was at the oyster-boat, buying his oysters, at three o'clock in the morning, wheeled them three miles, set up his board near a market, and began business. He sold out his oysters as fast as he could open them, at a good profit. He repeated this experiment morning after morning until he had saved $130, with which he bought a horse and wagon and had five cents left.

"How are you going to board your horse?" asked a stable-keeper, who witnessed this audacious transaction.

"I am going to board him at your stable."

"But you're a minor," replied this acute Yankee. "And mind, I can't trust you more than a week." The next morning the lad, who had established a good credit with the oyster-men, bought thirteen bushels of remarkably fine oysters, which he sold in the course of the day at a profit of seventeen dollars. So he was able to pay for his horse's board. And right there in the same market he continued to deal in oysters and fish for forty years, became king of that business, and ended by founding a college; thus affording a new illustration of Professor Agassiz's theory that the consumption of fish is serviceable to the brain.

So Astor, on reaching New York, with his capital of seven flutes and a few shillings, goes to work beating furs for two dollars a week, and keeps at furs until he is able to build Astor Houses and Astor Libraries. William Chambers, the founder of the great publishing house of Edinburgh, coming out of his apprenticeship at nineteen with five shillings capital, set up a bookstall with ten pounds worth of books, all bought on credit. The Harpers began by cautiously printing five hundred copies of "Locke on the Understanding," and Daniel Appleton by publishing a minute volume, bound in blue paper, two and a half inches square, called "Crumbs of Comfort." George Stephenson, brakeman to a steam engine at the mouth of a mine, began, it is true, by soling his sweetheart's shoes and demanding a kiss in payment. But this was only a youthful sally. Her name, however, was Ann, and she was a servant girl. But soon he began to tinker at his steam-engine, and kept on in that way until he invented the locomotive, and created, with the aid of his son, the railway system.

In those lecturing tours, which are far more instructive to me than I can be instructive to any one else, I frequently see immense establishments, and always visit them when I can. Nine times in ten, if I am told their history, I am informed that their founder was a poor man, who began business on next to nothing. In Chicago, a few years ago, a mechanic invested his whole capital, and credit too, in the making of one rough, strong farm wagon, the first ever made west of the lakes. It was all he could do to live while he made it, and if he had not had the good luck to sell it immediately he would have been in a sorry plight. When I was there, twenty years after, he had a factory which turned out an excellent wagon every seven minutes. Last winter, in Norwich, New York, I went over David Maydole's manufactory, where one hundred men were employed in making hammers. He is one of the most perfect examples of a king of business I have ever met with in my life. If every king of business were such as he, we should have the millennium the year after next. A plain little man he is, past sixty now, but in the full enjoyment of life, and in the full enjoyment of his work. Upon being introduced to him in his office, not knowing what else to say, and not being aware that there was anything to be said or thought about hammers—having, in fact, always taken hammers for granted—I said: "And here you make hammers for mankind, Mr. Maydole?"

"Yes," said he, "I've made hammers here for twenty-eight years."

"Well, then," said I, still at a loss for a talk-opener, "you ought to be able to make a pretty good hammer by this time."

"No, sir," said he, "I never made a pretty good hammer; I make the best hammer made in the United States."

And so he does; every hammer is made most carefully by hand, and tempered over a slow fire, as delicately as Delmonico's cook broils a steak for his pet gourmand. Then a hickory handle is put to it that has been seasoning for two years, and it is a hammer that dare show itself anywhere in the world. There is thought, and conscience, and good feeling, and high principle and business sense in it. It speaks its maker's praise wherever it goes and as long as it lasts, and it will last very long indeed. He did me the honor to give me one, which has ever since hung conspicuously in my room, admonishing me to work, not fast, nor too much, nor with a showy polish nor any vain pretence, but as well as I can every time, never letting one thing go till I have done all that was possible to make it what it should be.

Upon our return to the office he told his story. It is a representative story. Twenty-nine years ago, when he was a roadside blacksmith, six carpenters came to the village from the next county to work upon a new church, one of whom having left his hammer behind, came to the blacksmith's to get one made.

"Make me a good hammer," said the carpenter; "as good a one as you know how."

"But," said the young blacksmith, who had already considered hammers, and had arrived at some notion of what a hammer ought to be, and had a proper contempt for cheapness in all its forms, "perhaps you don't want to pay for as good a one as I can make."

"Yes, I do; I want a good hammer."

And so David Maydole made a good hammer—one that perfectly satisfied the carpenter. The next day the man's five companions came, each of them wanting just such a hammer, and when they were done the employer came and ordered two more. Next the storekeeper of the village ordered two dozen, which were bought by a New York tool merchant, who left a standing order for as many such hammers as David Maydole could make. And from that day to this he has gone on making hammers, until now he has one hundred and fifteen men at work. He has never advertised, he has never published, he has never borrowed. He has never tried to compete with others in price. He has never reduced a price because other men have done so. His only care has been to make a perfect hammer, to make as many such as people wanted and no more, and to sell them at a fair price.

THE SAME OLD STORY.

It is essentially the same story with all these kings of business. They learn how to do some one thing superlatively well, and then they keep on doing it better and better. Near Pittsburgh there is the great Cambria Iron Works, which employs seven thousand persons in making steel rails and iron—a great town of people, all in the service of one company. "What is the secret of such a development of business as this?" a visitor asked of the President and ruling spirit, Daniel J. Morrell. His answer was: "We have no secret. We always try and beat our last batch of rails. That's all the secret we've got, and we don't care who knows it."

In Philadelphia, Henry Desston & Sons sell five tons of saws every day—an immense quantity, for a saw is very thin and light. Forty years ago he landed on these shores, aged fourteen, with his father and sister, and two days after landing the father died, leaving those two orphans alone in a strange land. He got work in a saw-shop, and by-and-by began business for himself in a small cellar. The simple secret of his marvelous prosperity is that he studied saws to the very utmost, both theory and practice, and learned how to make better saws than had ever been made before.

Why are the Rothschilds the first bankers of the world? Because in a business career of one hundred and two years they have never failed to keep an engagement. Why is the Chemical Bank in New York the most solid and profitable bank in America? Because in the panic of 1837, when all other banks refused to pay gold for their notes, that bank did not, and never has. When gold was 286, if you presented one of its fifty-dollar notes at the counter and asked for its equivalent in gold, you got fifty dollars in gold. Why is the Ætna Insurance Company of Hartford the first of its kind in America? Simply because, after the great fires of New York, Portland, Chicago and Boston, it did what it had undertaken and engaged to do—paid its losses. When Cornelius Vanderbilt, at eighteen, learned that to him had been awarded the contract for conveying supplies to the different forts in New York harbor, he stared with astonishment. He had disdained to compete with the other boatmen in price, but had offered to do the work on just terms. The commissary, observing his surprise, said to him: "Don't you know why we have given this contract to you?" "No," replied the youth. "Why, it is because we want this business done, and we know you'll do it."

HONESTY THE ROCK—VANDERBILT'S RULES.

In the whole world I do not believe there can be found a business fifty years old which is not founded on the principle of rendering an equivalent for all that it receives. Honesty is the rock upon which all enduring success rests.

I was very much struck during the late panic with three rules which Vanderbilt gave to men in Wall street: "1. Never use what is not your own. 2. Never buy what you cannot pay for. 3. Never sell what you haven't got."

From what agonies of apprehension and remorse and shame men would be saved by the observance of these rules.

BRAIN CAPITAL.

Next to honesty I think we must place, as a condition of kingship in business, knowledge. I have observed that the men who take the lead in affairs, whatever else they lack, are sure to possess a most minute and entire knowledge of their branch of business—such a knowledge as can be got only by taking hold and doing every part of it. Girard, as sailor, mate and captain, had visited every port with which as a merchant he traded. He knew the men, the people, the markets. Astor, with a pack on his back, had tramped over the whole fur-producing region of New York and the lake country. No man ever knew furs as he knew them. He loved a fine fur as a connoisseur loves a fine picture. Stewart has the best touch for silk or velvet, and the best judgment of colors, of any man in his establishment. All the Harpers, young and old, began by setting type and working the press. The late John Walter, proprietor of the *London Times*, called at the office of the paper one day before going to the House of Commons, of which he was a member. While there a courier brought in a package labeled "Immediate and important," which he found to contain news of the greatest interest. It happened that all the compositors had gone to dinner. He took the dispatch up to the composing-room, set the type himself, and by the time the men came back he had it all ready to go into a second edition, which was immediately issued. He knew the business from top to bottom—knew it in his brain, and knew it in his fingers,

So Horace Greeley, on returning from his first visit to Europe, made up the steamer's news for the *Tribune* before she entered the harbor. The steamer arrived at six o'clock in the morning, after all the papers had been printed. Going straight to the office, he, too, found the compositors all gone home, and the pressman just preparing to go. He began forthwith to set the news in type, and he never left the case till it was all ready for an extra. Then he started up town to see his family. Old Jonas Chickering, old Mr. Steinway, could make a piano from the legs to the keys, every part outside and inside. The original Delmonico was himself an admirable cook. A thousand examples could be given showing that the capital of a house of business is not money, but brains.

SELF-CONTROL A NECESSITY.

Again: before a man can be a king of business or a king of men he must be monarch of himself. A great part of the secret of being able to control others is self-control. I remember Robert Bonner pointing out a person going by the office of the *Ledger*, and saying: "I worked by the side of that man for years setting type, and a very good workman he was. Do you want to know the reason why he is still a journeyman printer and I am not?" I did want to know the reason. "Well," said he, "the reason is this: he used to buy five dollar pantaloons, and as soon as they began to look shabby he cast them aside; but I bought coarse, strong, three dollar ones, and wore them out. That's the reason."

There is a great deal in merely being able to feel money in your pocket and not spend it. I must own that it is a very rare gift with the literary class. I have known a young writer, on receiving thirty dollars for an article, invite a friend to dine with him at Delmonico's and order two bottles of six dollar wine. Such men, whatever their talents, usually remain drudges and slaves all their lives. The simple reason, in fact, why property always and everywhere gets into such enormous masses, is that it is the nature of the strong to husband their resources and themselves, and it is the nature of the weak to squander both. If you want to test a young man and ascertain whether nature made him for a king or a subject, give him a thousand dollars and see what he will do with it. If he is born to conquer and command, he will put it quietly away till he is ready to use it as opportunity offers. If he is born to serve, he will immediately begin to spend it in gratifying his ruling propensity. That propensity may be, usually is, perfectly innocent. In my youth, for example, books were my temptation, and many a fierce tussle I have had with it while standing before the window of a bookseller. The first time in my life that I ever had two dollars, all at once, I instantly bought a Shakspeare with it. Knowing my weakness, I used to leave my money at home, when I had any, in order not to be surprised into buying a book; but feeling that this was base cowardice, a contemptible avoidance of the enemy, I afterward made it a point always to have money in my pocket. Often I have courted temptation, standing long before a window, gazing upon some particular book that I had been longing to possess for many months, and then stalking away with a proud consciousness that I might have bought it and did not. But, in my case, this was not strength, but mere vulgar necessity. The strong men are they who really might lawfully and properly indulge an expensive taste, and yet can wait till they can indulge it with absolute safety.

KINGS OF BUSINESS—KINGS OF MEN.

But all these qualities that I have mentioned—honesty, knowledge, self-control, resolution, perseverance—will not make a man a king of business. An individual, let him be the greatest man that ever lived, cannot accomplish much unless he knows how to avail himself of the services of others. I remember hearing Mr. Prang, the great chromo maker, say that the hardest thing he ever had to learn was to keep his own hands off the work, it was so much easier and quicker to take hold and do a difficult thing than to get another person to do it. But he soon found that the master of a large establishment must use all his skill and energy in doing just that, for it is only by doing nothing that he can do everything. A king of business is a king of men. He knows how men feel and think; what are their ruling motives and their disturbing foibles; where human nature is weak, where strong, and what makes men contented and discontented. He is a judge of men, and knows how to pick out the men he wants, and keeps them by treating them as he would like to be treated in their place.

Almost every day I pass the stage door of Wallack's Theater, in New York, and I notice there the same doorkeeper whom I saw on duty in the same situation when the father of the present Mr. Wallack opened the establishment twenty years ago. When I enter the theater in the evening I hand my ticket to the person who took my ticket from me on the opening night. I see there the same money-taker, ushers and actors, year after year—men grown gray in the service. In this fact we have a part of the reason why this theatre holds its own against all competition, and in spite of all changes in the public taste. Generally speaking, no business is more precarious than the theatrical, but this theater's business is as steady as that of an old bank. I have been there hundreds of times and never saw a non-paying house. The manager knows how to select the right men, and also how to make it their interest and their pleasure to remain.

SLAVES OF BUSINESS.

In our complicated modern life a man may get together an enormous mass of property, and yet never become a king of business. There are kings and there are slaves of business, and it is not till a man has made a fortune that we can certainly tell to which of these classes he belongs.

It was said during the late panic of an enormously rich man, who makes great pretensions to religion, that he could lose three millions of greenbacks without feeling it, but the loss of five dollars worth of his religion would bankrupt him. He was a slave of business, inasmuch as he regarded the accumulation of money as an end and not as a means. He valued his five millions only as a means of getting another five. No man is such a slave as one in whom the gentler affections are dead, and who lives only to accumulate property. The late Edwin Forrest was a melancholy instance of this. He used to din in the ears of his patriotic and generous friend, Murdoch: "Work, work while you can; money is power." It was a strange mistake for such a man, who had peculiar experience of the powerlessness of money to bring into a human life one gleam of joy. Other men, generous and good, get drawn into a roaring whirlpool of business, from which they have not the strength to escape, not the less are slaves of business. I heard one of these some time ago relate how he became a slave, and how at last he escaped from slavery. At the age of twenty-eight he began making iron and steel in Pittsburg—made $1,000,000 worth per annum upon a capital of $150,-000. It was that absurd disproportion between capital and production which made him a slave, always anxious, often in terror, always overworked. For fourteen years he toiled eighteen hours a day, and hardly had a personal acquaintance with his own children. At length the victory was won. He had a capital of $3,000,000, twenty-five hundred men employed, the finest works in America,—a little town of brick cottages—and his paper gilt-edged. But he had now become the slave of this vast business, because he had not taken care to train and form men to do the difficult part of the work, finding it easier to do it himself, as it was for the moment. So he kept on, called every morning at six, away to the store before seven, in the counting-room till nine, then to the works, where he remained till two. After hasty dinner he hurried away to the mine, getting home to tea at dark. After tea, more dead than alive, he would drag around to his office and estimate till midnight. Then to bed and instantly to sleep. But, one night after such a day of toil—three fair days' work in one—sleep came not to his eyelids for hours, and when it did come it was not the deep sleep to which he was accustomed. Many such nights followed. Then one morning, while he was in the midst of an abstruse calculation, suddenly his brain lost its grasp of the problem. He could not fix his attention upon it. He forgot the early steps of the process. He was obliged to give it up. A short journey seemed to restore his mental power; but a few days after his return, at the same hour—eleven in the morning—again he lost it. He tried to think of some way of amusing himself for an hour or two, but he could think of nothing but to go to the dentist's and enjoy a little refreshing agony. He failed in this attempt to obey his physician, for while waiting in the dentist's ante-room he fainted, and had to be carried home. Now, for the first time, he took his case into as serious consideration as he had been accustomed to bestow upon iron. He studied the brain as if it had been a new kind of steel. He saw his error, and, what is much more difficult and unusual, he reformed his life. He became intimately acquainted with his children, spent every evening at home, took time for any innocent pleasure that fell in his way, had a joyous holiday in summer, bought a nice pair of horses, drove them every fine day, and so gradually recovered his health. I caught sight of him the last time I passed through smoky Pittsburgh, seated in a light wagon, looking very well—a happy, broad-chested king of iron.

But for one man who has the resolution to conquer a fixed habit of overwork, ten die of it in the midst of their days. It is not, I repeat, till a fortune is won that we can tell whether a man is master or slave; and this is particularly true of the present time, when a mere flash-in-the-pan like Fisk can get control sometimes of large masses of property. Of our rich men we can say, by their hobbies shall ye know them. A few of the stupidest and vulgarest destroy themselves by luxurious living. Many destroy their children by indulgence and neglect. We had a case in New York during the war of a father who gave his son, aged twenty, one hundred dollars every Monday morning. Nothing, indeed, tests a rich man's manhood so much as the manner in which he deals with his children. To make a fortune is not easy, but to bring up a child fit to inherit it is very difficult. Here is the touchstone which indicates with almost unerring certainty whether a man is master or servant of his fortune. Is he master? Then he uses his wealth to make it a good to his children. Is he slave? Then he permits his wealth to effeminate and sensualize them, to make them vain, selfish and helpless. A man must be exceptionally wise and strong who, in the compass of one short lifetime, can both acquire riches and learn the difficult art of being rich without doing his family any harm by it.

THE FOOLISHNESS OF THE RICH.

By their hobbies shall ye know them. The favorite hobby at present of the wealthiest slaves of business is to give away or bequeath stunning sums of money for unnecessary or impossible objects—imposing upon posterity tasks that will not be performed. The thoughtless praise lavished upon such people as Girard ought not to mislead us as to the real merits of the men and the true character of their acts.

The wisest and greatest man that ever lived could scarcely, even if he were perfectly unshackled, execute such a will as Girard's without doing more harm than good. But that huge legacy, now worth $30,000,000, has been administered by the gang of pot-house politicians who for the past thirty years have constituted the government of Philadelphia. If Girard, during the last year of his life, had loaded one of his ships with all that gold scraped together by fifty years of miserly solicitude, and poured it out into the unfathomable sea, he would have rendered a better service to Philadelphia than he did by leaving it to found an orphan asylum on a scale far beyond the wit of mortals to conduct successfully—a huge boarding school of a thousand pupils. There was a printer in New York who took it into his head to raise chickens on Staten Island for the New York market. He bought a farm, fenced it in, and began with a small family of two thousand chickens. There never was known such a time among the farmers' wives of Staten Island for selling off their old hens.

He had beautiful contrivances for feeding, watering and sheltering his numerous flock; patent nests, convenient egg receptacles, and every device of the chicken farmer. But, for some reason unknown, scarcely any eggs appeared, few chickens were hatched, the birds pined and drooped, and soon so many dead ones strewed the ground of a morning that they had to be collected in a wheelbarrow—the dead-cart of this chicken city. In short, he discovered that chickens cannot be raised thousands in a family. They will not thrive in masses. Nor will children. You could only have a beneficial orphan asylum on that scale by making an artificial village, with its schools, and the boys divided into groups as closely as possible resembling families. How can the Girards of the world, men who live without love, upon whose knees children never sit, who repel and drive far from their hearts and homes their own kindred, who know nothing of any kind of power except that which is connected with the signing of checks; hard men, ignorant of every phase of human existence except banking and stocks, how can such people be rationally expected to create institutions the most complicated, difficult and delicate known to civilization? I once heard from the lips of Girard's lawyer, the late William J. Duane, of Philadelphia, a description of the scene that occurred in Girard's house after his death; Mr. Duane was executor, being in charge of the property. As soon as the breath was known to be out of the old man's body, and Mr. Duane had closed his eyes, it seemed as if the spell had been suddenly dissolved, and the numerous nephews and nieces and their descendants, who never before had stood in Girard's presence but with fear and trembling, burst into exultation. A fierce joy shone in every face. The younger men rushed down into the cellar and brought up bottles of their uncle's choicest wine, hoarded there for years, of which they had never been invited to taste. Some of them were far gone in intoxication before the body was cold. Older men rummaged the rooms; women searched the closets and drawers. The whole house was a scene of wild riot. They behaved, in fact, like a select party of vultures, which from a safe distance have followed and watched a sick buffalo, and when at last the monarch of the prairie droops, lies down and falls over upon his side a dead creature, then they swoop down from the sky and pick out his eyes, tear out his vitals, and shriek exultant as they do it, each foul bird glaring hate upon the rest, and devouring with his vulture eyes the whole carcass. When they had ranged all over the house, they came in a body to Mr. Duane and demanded to know if there was a will. There was. He had drawn it himself two years before. It was in the iron safe in the room where the dead man lay. Upon hearing this a frenzy of desire seized them to know its contents, and they insisted on hearing it read then with such infuriate clamor that Mr. Duane, knowing how the will would avenge his client and rebuke this inhuman indecency, consented at length to read it, and it was read.

"When I had opened the will," said Mr. Duane to me, "and was about to begin to read, I chanced to look over the top of the document to the company seated before me. It was a sight never to be forgotten. There was a ghastly pallor on every face, and a certain look of mingled curiosity, greediness and jealousy, which I am sure the greatest artist that ever lived could not have done justice to. Years have gone by, and I can see it still."

He began to read. When it became evident that the bulk of the estate was left for public objects, the affectionate relations, who had been left only a few thousands each, made not the least pretence of concealing their rage; and, as you know, they spent their legacies in vain attempts to break the will. Unloved in their lives, unblest in death are such slaves of business as Stephen Girard. They are foolish to make so much money, they are foolish to leave it for objects of which they know less than nothing, and the public is not wise in accepting their gifts.

Once already within the historic period Christendom has been cursed with institutions founded by mistaken benevolence—convents and monasteries—which cost nations a revulsion to suppress. Let us beware of repeating the error.

### BEN FRANKLIN A MASTER OF BUSINESS.

In that same city of Brotherly Love there once lived a perfect king of business, a noble contrast to this barren, blind, well-intentioned Girard. I mean the generous, the genial, the wise, the public-spirited Franklin, who knew precisely what money can do and what it cannot do. At the age of forty-two, when he had been in business twenty years, he had the most lucrative and famous publishing and printing house in all the colonies. His *Gazette* was the best and most profitable of American newspapers; his comic almanac, called "Poor Richard," was the most popular publication of the kind in the world; he was printer to four colonial governments; he had an extensive business in books, stationery, rags, lampblack and ink. He had but to keep on to make a ridiculously great fortune, and become a slave to business. But having acquired an estate of about £700 a year, and being deeply interested in science, he deliberately resolved to retire and dedicate the rest of his life to the increase of knowledge. He sold out his business on easy terms to his foreman, and gave himself up to electricity, from which, however, he was soon drawn away into public life. Twenty years he spent in acquiring independence. Then he gave the young men a chance to do the same. The remaining forty-two years of his happy and victorious existence he employed in the service of his country. Business was his servant, never his master. Make money, therefore, by any honorable means within your reach and your ability. But when you have won independence, withdraw and make room for younger men. Then set up, not a carriage merely—any one can do that—but a hobby, suited to your powers and means. Choose it well. If you have a turn for improving the breed of sheep or pigs, do that. If you dote upon a horse, raise a colt to beat Dexter. If it is in you to write a book, write it. If science attracts you, pursue your favorite branch. If you hate to see half the people here living—no, dying—in tenement houses, widen the island by setting on foot a system of bridging and tunneling the rivers, and killing the fever and ague in Westchester county and Staten Island. If public life and immortal fame allure you, run for Congress, and carry international copyright. If nature has gifted you with social talents and a friendly heart, throw open wide your house and entertain people rationally and handsomely. If the sorrows of the poor and the orphan move you, pass happy days in helping them help themselves. If you desire to found an institution, do it. Don't hire it done after your death. If you bequeath money for public objects, give it without conditions to an institution which has shown some right to exist by having existed. Then may it be written upon your gravestone, or your urn, if you prefer cremation: "This person, having fairly won the right to his time, set up a noble hobby and rode it worthily all the rest of his days."

